# Presynaptic neuromuscular action of a methanolic extract from the venom of *Rhinella schneideri* toad

**DOI:** 10.1186/1678-9199-20-30

**Published:** 2014-07-04

**Authors:** Sandro Rostelato-Ferreira, Cháriston A Dal Belo, Gildo B Leite, Stephen Hyslop, Léa Rodrigues-Simioni

**Affiliations:** 1 Departamento de Farmacologia, Faculdade de Ciências Médicas, Universidade Estadual de Campinas (UNICAMP), CP 6111, Campinas, SP 13083-970, Brasil; 2 Laboratório de Neurobiologia e Toxinologia, (LANETOX), Universidade Federal do Pampa, (UNIPAMPA), Av. Antônio Trilha, 1847, Centro, CEP 97300-000 São Gabriel, RS, Brazil

**Keywords:** Neurotransmitter release, Ouabain, Presynaptic, *Rhinella schneideri*, Toad venom

## Abstract

**Background:**

*Rhinella schneideri*, previously known as *Bufo paracnemis,* is a common toad in many regions of Brazil. Its venom exerts important cardiovascular effects on humans and other animals. Although this toad venom has been the subject of intense investigations, little is known about its neuromuscular activity.

**Methods:**

The neurotoxicity of a methanolic extract of *R. schneideri* venom was tested on mouse phrenic nerve-diaphragm (PND) preparations mounted for conventional twitch tension recording – in response to indirect stimulation – and for electrophysiological measurements.

**Results:**

Venom extract (50 μg/mL) increased the muscle twitch tension in PND preparations but did not significantly alter the resting membrane potential values. Electrophysiological evaluations showed that the extract (50 μg/mL) significantly augmented the frequency of miniature end-plate potential (from 38 ± 3.5 to 88 ± 15 after 60 minutes; n = 5; *p* < 0.05) and quantal content (from 128 ± 13 to 272 ± 34 after five minutes; n = 5; *p* < 0.05). Pretreatment with ouabain (1 μg/mL) for five minutes prevented the increase in quantal content (117 ± 18 and 154 ± 33 after five and 60 minutes, respectively).

**Conclusion:**

These results indicate that the methanolic extract of *R. schneideri* venom acts primarily presynaptically to enhance neurotransmitter release in mouse phrenic-diaphragm preparations.

## Background

Amphibians produce cutaneous secretions that serve as a defense against predators [1]. Toads in particular have highly toxic venom that is produced by well-developed postorbital parotid glands [2]. The cardiovascular effects of toad venom on vertebrates are well known and have been extensively investigated, particularly in the genus *Rhinella*[3,4]. The main venom toxins responsible for its cardiac effects are bufadienolides and bufotoxins that increase cardiac contractility and decrease cardiac rate by inhibiting the Na^+^/K^+^-ATPase pump in a manner similar to digoxin [3,4]. In contrast, little is known about the neuromuscular activity of toad venoms, although it was previously observed that envenoming in dogs may be accompanied by neurological manifestations such as mydriasis, nystagmus and opisthotonus [5].

*Rhinella schneideri* (formerly known as *Bufo paracnemis* Lutz, 1925) is a common toad in several South American countries [6]. A previous study observed that rats injected intraperitoneally with *R. schneideri* venom (2–5 mg) show uncoordinated movements, dyspnea, convulsions and paralysis, followed by respiratory and cardiac arrest [7]. This sequence of events indicates that in rats neurotoxic manifestations precede cardiac effects, an order that is peculiar to this venom. In the present work, we examined the neurotoxicity of a methanolic extract obtained from Brazilian *R. schneideri* venom on mouse neuromuscular preparations *in vitro*.

## Methods

Venom was collected by manual compression of large postorbital parotid glands from two toads. An amount of 2 g was then extracted with methanol (50 mL) for three days at room temperature, after which the resulting extract was lyophilized in a SpeedVac® centrifuge (Savant, USA) [8]. The methanolic extract was lyophilized and dissolved in Tyrode solution prior to testing on neuromuscular preparations.

Male Swiss white mice (25–30 g) were obtained from the Multidisciplinary Center for Biological Investigation (CEMIB/UNICAMP). The animals were housed at 23 ± 3°C on a 12-hour light/dark cycle with free access to food and water.

The diaphragm and its phrenic nerve were dissected from male Swiss mice killed with isoflurane (Cristália, Brazil). The preparations were mounted under a resting tension of 5 g in a 5 mL organ bath containing aerated (95% O_2_ and 5% CO_2_) Tyrode solution (composition, in mM: NaCl 137, KCl 2.7, CaCl2 1.8, MgCl2 0.49, NaH2PO4 0.42, NaHCO3 11.9 and glucose 11.1) at 37°C, as described by Bülbring [9]. Supramaximal stimuli (0.1 Hz and 0.2 ms for indirect stimulation) were delivered from a Grass S88 stimulator (Grass Instrument Co., USA) and the muscle twitches were recorded using a Load Cell BG-25 g force displacement transducer coupled to a Gould RS 3400 recorder (both from Gould Inc., USA). The preparations were allowed to stabilize for at least 20 minutes before the addition of methanolic extract (50 μg/mL).

End-plate potentials (EPPs), miniature end-plate potentials (MEPPs) and resting membrane potentials (RPs) were measured with a high input impedance electrometer (World Precision 750, USA) in mouse diaphragm muscle preparations using conventional microelectrode techniques. The dissected muscle was mounted in a lucite chamber containing aerated (95% O_2_ and 5% CO_2_) Tyrode solution (pH 7.4, at room temperature of 23-27°C) with or without methanolic extract (50 μg/mL). Intracellular microelectrodes filled with 3 M KCl (resistance 15–25 MΩ) were used. The EPPs, MEPPs and muscle RPs were recorded on an oscilloscope (Tektronix, USA) and subsequently documented as described below. The RP recordings were taken at the end-plate regions in the absence or presence of methanolic extract at t0 (basal), t5, t15, t30 and t60 minutes.

EPPs were recorded in muscles previously subjected to the cut muscle technique in order to uncouple muscle contractions from nerve stimulation. [10]. A direct-current channel was used to record the RPs and an alternate-current channel was used to record the EPPs. The EPPs were magnified (AM 502 Tektronix amplifier, gain ¼ 100), low-pass filtered (3 kHz) and digitized (15 kHz sampling rate) using an analog-to-digital converter (Lynx, Brazil; CAD12/36, resolution: 12 bits) coupled to a microcomputer (Microtec, Brazil) loaded with AqDados 5 software (Lynx) that enabled digital storage of the EPPs online and their subsequent retrieval for measurement and analysis.

For measurement of the quantal content of EPPs, a stimulus rate of 1 Hz for one minute was generated at t0 (basal), t5, t15, t30 and t60 minutes and 30 to 60 potentials were measured at each interval. The quantal content (QC) was estimated as the quotient between the squared average of the EPPs and the variance of the EPPs (indirect method), as described by Dal Belo *et al.*[11].

MEPPs were recorded in uncut muscle without generating electric stimuli. MEPP measurements were obtained before (t0) and at various intervals (t5, t15, t30 and t60) after methanolic extract addition.

Each experimental protocol was repeated 3 to 8 times and the results were reported as the mean ± S.E.M. Student’s *t*-test and repeated-measures analysis of variance (ANOVA) were used for statistical comparison of the data, with a value of *p* < 0.05 indicating significance. All data analyses were done using OriginPro 8.

## Results and discussion

**Figure 1:**
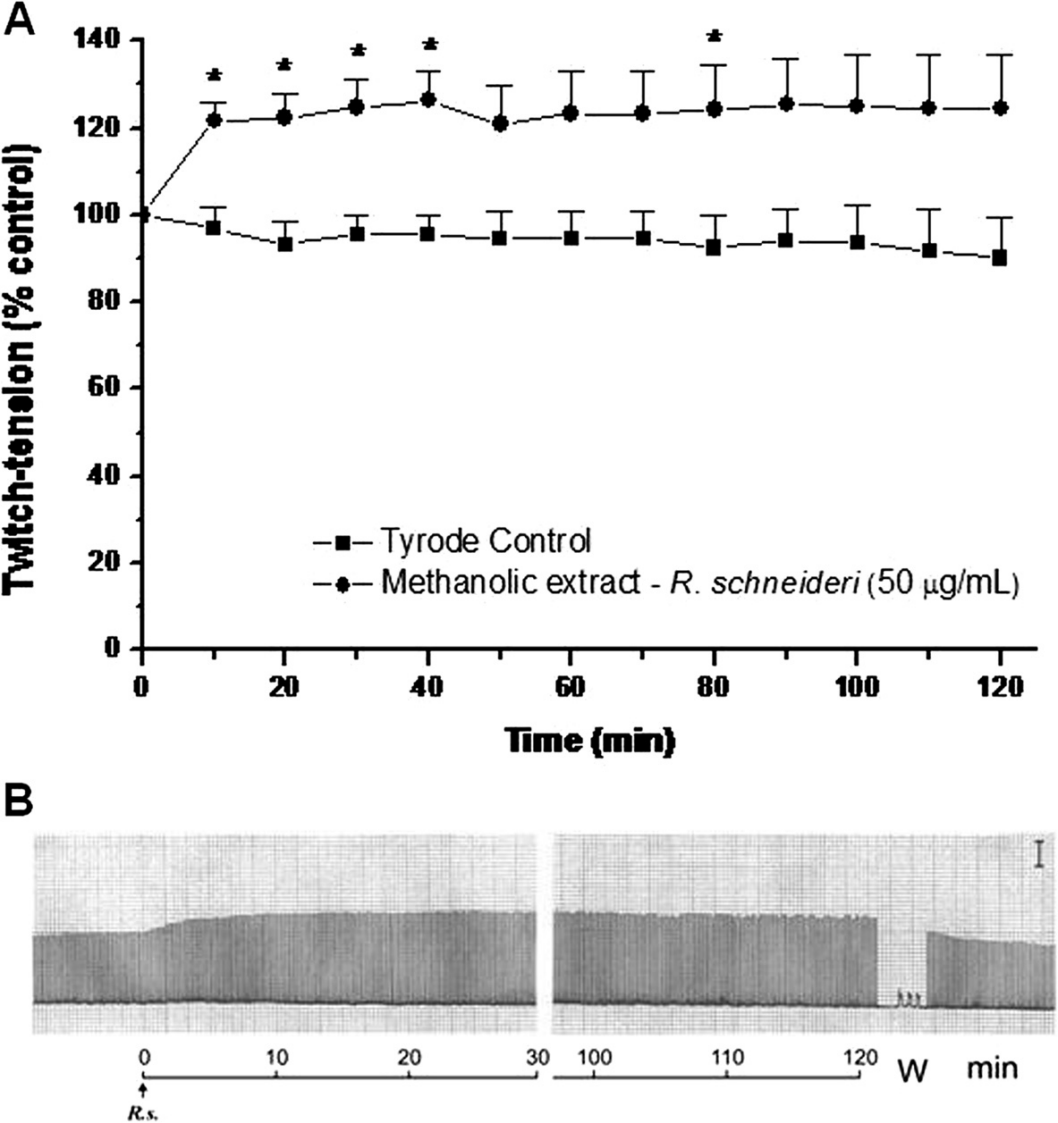
**Neuromuscular activity of methanolic extract of *R. schneideri *in vertebrate nerve–muscle preparations. (A)** Neuromuscular response produced by methanolic extract in indirectly stimulated mouse PND preparations. **(B)** Individual myographic record. Note the facilitatory response without neuromuscular blockade. (W, wash; Bar: 5 g). The points represent the mean ± S.E.M. of 5–6 experiments. *p < 0.05 compared to the twitch-tension at time 0.

The methanolic extract (50 μg/mL) produced an increase in muscle twitch tension in PND preparations (Figure 1) during a 120-minute observation. There was little concentration dependence in this effect since the responses at lower (25 μg/mL) and higher (200 μg/mL) concentrations of extract were not significantly different from those observed with 50 μg/mL (data not shown).

**Figure 2:**
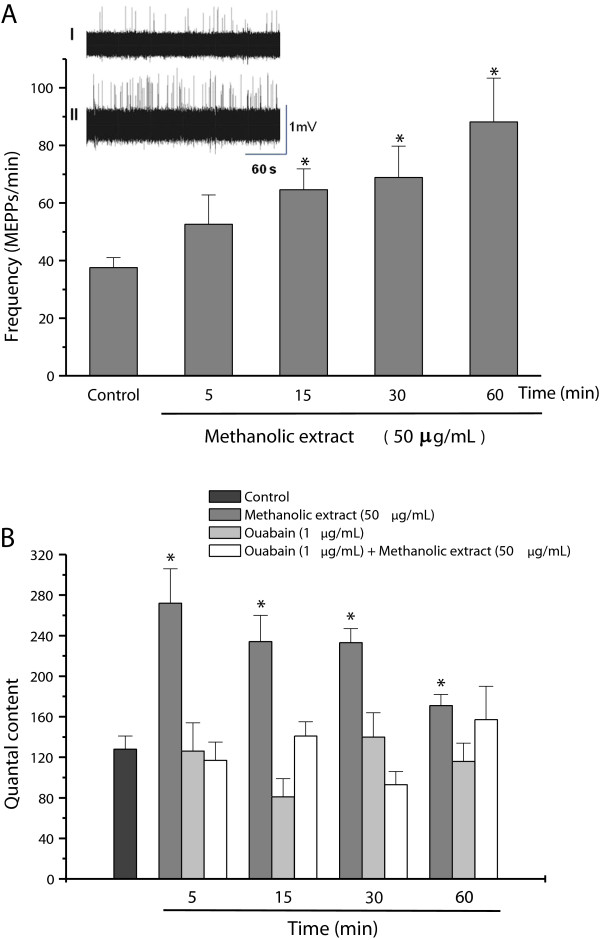
**Electrofisiological measurements in PND preparations. (A)** Changes in the frequency of miniature end-plate potentials (MEPPs) in phrenic nerve-diaphragm preparations incubated with a methanolic extract of *R. schneideri* venom (50 μg/mL). Insets I and II in **A**: recordings at time zero (0) and after incubation with extract for 60 minutes, respectively. **(B)** Changes in the quantal content (end-plate potentials) of phrenic nerve-diaphragm preparations incubated with a methanolic extract of *R. schneideri* venom (50 μg/mL) for up to 60 minutes and the effect of pretreatment with ouabain (1 μg/mL). The columns are the mean ± S.E.M. (n = 5), *p < 0.05 compared to control preparations.

The methanolic extract (50 μg/mL) significantly increased the frequency of MEPPs from 38 ± 3.5/minute (control) to 88 ± 15/minute after 60 minutes (Figure 2A), without affecting the amplitude of these potentials (data not shown). Incubation with extract resulted in a significant increase in the quantal content within five minutes followed by a gradual decrease towards basal (control) values over the next 60 minutes, although the level after 60 minutes was still significantly greater than the control (Figure 2B). Pretreatment of the preparations with a non-toxic concentration of ouabain (1 μg/mL), an inhibitor of Na^+^/K^+^-ATPase pump activity, for five minutes prior to incubation with the methanolic extract prevented the increase in quantal content (Figure 2B), although ouabain alone had no effect on this parameter [12].

Incubation with the methanolic extract (50 μg/mL) did not affect the resting membrane potential of the preparations [81 ± 0.9 mV (control) vs. 78 ± 0.9 mV after 60-minute incubation with extract].

Rostelato-Ferreira *et al*. [13] employed the methanolic extract (3–30 μg/mL) on chick biventer cervicis preparations and observed a concentration-dependent neuromuscular blockade that was preceded by significant facilitation of neurotransmission at 10 μg/mL. However, this response was not accompanied by alterations in muscle contractures to exogenous ACh or KCl, indicating that the extract had no inhibitory effect on postsynaptic nicotinic receptors and neither interfere with the muscle contracture mechanism.

The results described herein show that a methanolic extract from *R. schneideri* venom contains substances capable of affecting neurotransmission in PND preparations. The extract caused sustained muscle facilitation that apparently resulted from enhanced presynaptic neurotransmitter release since electrophysiological measurements indicated an increase in MEPP frequency; such a response is generally associated with a facilitatory effect on neuromuscular transmission [14,15]. In contrast, variations (increase or decrease) in MEPP amplitude – such as those that occur with curare and anticholinesterase drugs – are indicative of a postsynaptic action [16]. The absence of these variations in PND preparations incubated with the extract suggested that there was no postsynaptic action involved. Further evidence for a presynaptic involvement was the marked increase in quantal content within five minutes after the extract addition to the preparations. Dal Belo *et al.*[11] observed a similar increase in the quantal content of PND preparations after ten-minute incubation with MiDCA1, a toxin isolated from coral snake (*Micrurus dumerilli carinicauda*) venom, and concluded that a presynaptic action was involved.

Digoxin, a cardiac glycoside, and ouabain, a cardiotonic steroid derivative structurally similar to digoxin, potentiate cardiac contractions by inhibiting the Na^+^-K^+^-ATPase pump in cardiomyocytes which, in turn, leads to the accumulation of intracellular calcium through indirect blockade of the Na^+^/Ca^2+^ antiport system. Low concentrations of these substances facilitate the spontaneous and evoked release of acetylcholine, thereby increasing the frequency of MEPPs, the amplitude of single end-plate potentials and their quantum content [17,18].

Toad venoms are highly cardiotoxic and exert their cardiotoxicity by a digoxin-like effect [19,20]. Marinobufagenin, an extensively studied component of *Rhinella marina* (previously *Bufo marinus*), is a specific inhibitor of Na^+^-K^+^-ATPase that acts through the cardiac glycoside binding site of this pump [21]. The experiments with ouabain described herein showed that the presynaptic effect of the methanolic extract was associated with the inhibition of Na^+^-K^+^-ATPase since preincubation with ouabain prevented the release of acetylcholine (increase in quantal content) in mouse PND preparations.

Previous reports have demonstrated that the quantal release of neurotransmitters from nerve terminals may be controlled by a ouabain-sensitive mechanism not directly related to the pump function of Na^+^-K^+^-ATPase [22]. On the other hand, ouabain-sensitive neuronal Na^+^-K^+^-ATPase apparently plays an important role in the frequency-dependent modulation of the quantum release of neurotransmitters [12]. Our experiments showed that the presynaptic effect of the extract was inhibited by a non-toxic concentration of ouabain (1 μM). While this finding suggests the involvement of Na^+^-K^+^-ATPase in this presynaptic action, the experimental protocol used here does not exclude the possibility of a non-specific interaction between ouabain and components of the methanolic extract. Albeit the blockade of the Na^+^ pump by a non-toxic concentration of ouabain (1 μM) is insufficient to impair the intracellular ionic homeostasis at low frequencies of stimulation, and the precise role of the Na^+^-K^+^-ATPase pump in the cholinergic modulation of presynaptic functions is still unclear [23]. Krivoi [24] suggested that activation of the Na^+^-K^+^ electrogenic pump could hyperpolarize nerve endings, thereby decreasing repolarization of the K^+^ current in presynaptic spikes and weakening the inactivation of sodium channels.

## Conclusion

In conclusion, our results suggest that the methanolic extract of *R. schneideri* venom acts primarily presynaptically to enhance neurotransmitter release in mouse phrenic-diaphragm preparations and these effects were prevented through the inhibition of neuronal Na^+^-K^+^-ATPase.

### Ethics committee approval

The present study was approved by the institutional Committee for Ethics in Animal Use (CEUA/UNICAMP, protocol no. 1552–1) and was performed in accordance with the ethical guidelines established by the Brazilian Society of Laboratory Animal Science (SBCAL, formerly the Brazilian College of Animal Experimentation – COBEA).

## Competing interests

The authors declare that there are no competing interests.

## Authors’ contributions

SRF and LRS are the lead researchers of this study. SRF, CADB and LRS designed the study. SRF and GBL performed experiments and their analysis. SRF, CADB, SH and LRS participated in the analysis of the results and article writing. All authors read and approved the final manuscript.
